# Differences in clinical outcomes of bloodstream infections caused by *Klebsiella aerogenes*, *Klebsiella pneumoniae* and *Enterobacter cloacae*: a multicentre cohort study

**DOI:** 10.1186/s12941-024-00700-8

**Published:** 2024-05-06

**Authors:** Mariana Guedes, David Gathara, Inmaculada López-Hernández, Pedro María Martínez Pérez-Crespo, María Teresa Pérez-Rodríguez, Adrian Sousa, Antonio Plata, Jose María Reguera-Iglesias, Lucía Boix-Palop, Beatriz Dietl, Juan Sevilla Blanco, Carlos Armiñanzas Castillo, Fátima Galán-Sánchez, Clara Natera Kindelán, Alfredo Jover-Saenz, Josune Goikoetxea Aguirre, Ana Alemán Alemán, Teresa Marrodán Ciordia, Alfonso del Arco Jiménez, Jonathan Fernandez-Suarez, Luis Eduardo Lopez-Cortes, Jesús Rodríguez-Baño, PROBAC/GEIRAS-SEIMC/SAMICEI group, Eva Leon, Inés Pérez Camacho, David Vinuesa García, Jordi Cuquet Pedragosa, Isabel María Reche Molina, Alberto Bahamonde-Carrasco, Carmen Herrero Rodríguez, Marcos Guzmán García, Antonio Sánchez-Porto, Alejandro Smithson Amat, Esperanza Merino de Lucas, Jesús Canueto Quintero

**Affiliations:** 1 Unidad Clínica de Enfermedades Infecciosas y Microbiología, Departamento de Medicina, Instituto de Biomedicina de Sevilla (IBiS)/CSIC, Hospital Universitario Virgen Macarena, Universidad de Sevilla, Seville, Spain; 2 Infection and Antimicrobial Resistance Control and Prevention Unit, Hospital Epidemiology Centre, Centro Hospitalar Universitário São João, Porto, Portugal; 3 London School of Hygiene and Tropical Medicine, MARCH Centre, London, UK; 4 CIBERINFEC, Instituto de Salud Carlos III, Madrid, Spain; 5 Unidad de Enfermedades Infecciosas y Microbiología, Hospital Universitario Virgen de Valme, Seville, Spain; 6 Unidad de Enfermedades Infecciosas, Departamento de Medicina Interna, Complexo Hospitalario Universitario de Vigo/Galicia Sur Health Research Institute, Vigo, Spain; 7 Servicio de Enfermedades Infecciosas, Hospital Regional Universitario de Málaga, IBIMA Málaga, Málaga, Spain; 8 Servicio de Enfermedades Infecciosas, Hospital Universitari Mútua de Terrassa, Barcelona, Spain; 9 Unidad de Enfermedades Infecciosas y Microbiologia Clinica, Hospital Universitario Jerez De La Frontera, Jerez De La Frontera, Cádiz, Spain; 10 Servicio de Enfermedades Infecciosas, Hospital Universitario Marqués de Valdecilla-IDIVAL, Universidad de Cantabria, Santander, Spain; 11 Unidad de Gestión Clínica de Microbiología, Hospital Universitario Puerta del Mar, Cádiz, Spain; 12 Unidad de Enfermedades Infecciosas, Hospital Universitario Reina Sofia, Cordoba, Spain; 13 Unidad de Infección Nosocomial, Hospital Universitario Arnau de Vilanova, Lleida, Spain; 14 Unidad de Enfermedades Infecciosas, Hospital Universitario de Cruces, Barakaldo, Spain; 15 Unidad de Gestión Clínica de Enfermedades Infecciosas, Hospital Universitario de Burgos, Burgos, Spain; 16 Departamento de Microbiología Clínica, Complejo Asistencial Universitario de León (CAULE), León, Spain; 17 Grupo Enfermedades Infecciosas, Servicio de Medicina Interna, Hospital Costa del Sol, Marbella, Spain; 18 Unidad de Microbiología, Instituto de Investigación Sanitaria del Principado de Asturias (ISPA), Hospital Universitario Central de Asturias, Oviedo, Spain

**Keywords:** Bloodstream infection, *Klebsiella aerogenes*, *Enterobacter cloacae*, *Klebsiella pneumoniae*, Mortality, Recurrence

## Abstract

**Background:**

*Klebsiella aerogenes* has been reclassified from *Enterobacter* to *Klebsiella* genus due to its phenotypic and genotypic similarities with *Klebsiella pneumoniae*. It is unclear if clinical outcomes are also more similar. This study aims to assess clinical outcomes of bloodstreams infections (BSI) caused by *K. aerogenes, K. pneumoniae* and *Enterobacter cloacae*, through secondary data analysis, nested in PRO-BAC cohort study.

**Methods:**

Hospitalized patients between October 2016 and March 2017 with monomicrobial BSI due to *K. aerogenes*, *K. pneumoniae* or *E. cloacae* were included. Primary outcome was a composite clinical outcome including all-cause mortality or recurrence until 30 days follow-up. Secondary outcomes were fever ≥ 72 h, persistent bacteraemia, and secondary device infection. Multilevel mixed-effect Poisson regression was used to estimate the association between microorganisms and outcome.

**Results:**

Overall, 29 K*. aerogenes*, 77 *E. cloacae* and 337 K*. pneumoniae* BSI episodes were included. Mortality or recurrence was less frequent in *K. aerogenes* (6.9%) than in *E. cloacae* (20.8%) or *K. pneumoniae* (19.0%), but statistical difference was not observed (rate ratio (RR) 0.35, 95% CI 0.08 to 1.55; RR 0.42, 95% CI 0.10 to 1.71, respectively). Fever ≥ 72 h and device infection were more common in *K. aerogenes* group. In the multivariate analysis, adjusted for confounders (age, sex, BSI source, hospital ward, Charlson score and active antibiotic therapy), the estimates and direction of effect were similar to crude results.

**Conclusions:**

Results suggest that BSI caused by *K. aerogenes* may have a better prognosis than *E. cloacae* or *K. pneumoniae* BSI.

**Supplementary Information:**

The online version contains supplementary material available at 10.1186/s12941-024-00700-8.

## Background

Bloodstream infection (BSI) is one of the infectious syndrome with the highest health burden and is associated with important costs to the healthcare system, [1–4]and in the last years the incidence of *Enterobacterales* BSI has been increasing [5–13].

Studies estimate that overall mortality in *K. pneumoniae* BSI can range from 20 to 54%, being particularly high in KPC-producing *K. pneumoniae* infections [12, 14–20]. *Enterobacter cloacae* BSI mortality seems to be lower than *K. pneumoniae*, ranging from 19 to 33%, [14, 21–24] with a risk of recurrence between 11 to 21%, higher in cases with extended-spectrum beta-lactamase (ESBL) production [22, 24–26]. Most of the studies report *K. aerogenes* outcomes aggregated in *Enterobacter* genus. Though, *K. aerogenes* BSI mortality appears to be lower than in *Enterobacter spp.* infections, between 10 and 21% [21, 27].

Since 1971, taxonomic studies have suggested that *Enterobacter aerogenes* should be classified into *Klebsiella* genus, due to its phenotypic and genotypic similarities to *K. pneumoniae* [28–31]. Recently, Tindall et al. has proposed to officially change its name to *K. aerogenes* and Clinical and Laboratory Standards Institute (CLSI) and European Committee on Antimicrobial Susceptibility Testing (EUCAST) have implemented it in their official documents [32, 33]. Genomic data also suggest that *K. aerogenes* could be more virulent than *E. cloacae*, since it possess virulence-encoding genes that were also identified in *K. pneumoniae*, but not described in *E. cloacae* [34–36]. If these similarities between *K. aerogenes* and *K. pneumoniae* translate into similar clinical outcomes is still to be determined. Some studies had tried to compare outcomes in *K. aerogenes* and *E. cloacae* BSI, but their findings have been contradictory and inconclusive [37–40]. We didn’t find any study comparing clinical outcomes in *K. aerogenes* and *K. pneumoniae* BSI.

This study aims to assess the clinical outcomes in patients with BSI caused by *K. aerogenes, K. pneumoniae* and *E. cloacae,* by exploring the association between a clinical composite outcome (mortality or recurrence) and these pathogens.

## Methods

### Study design

A secondary data analysis, nested in the PRO-BAC observational cohort study was performed. PRO-BAC was a Spanish multicentre study that prospectively included episodes of microbiological confirmed bacteraemia in hospitalised patients between 1st October 2016 and 31st March 2017 (Clinicaltrials.gov NCT03148769; Register date 05/04/2017) [41]. Screening was done by daily review of blood culture results; individuals without systemic signs or symptoms of infection and subsequent episodes caused by the same microorganism within three months of the primary episode were also excluded [41]. Microorganism identification and susceptibility testing was performed according to the local laboratory guidelines that were aligned with the 2016 EUCAST recommendations or the CLSI guidelines. Clinical outcomes such as fever ≥ 72 h, complications secondary to treatment and metastatic infections were recorded at 30-days follow-up after bacteraemia diagnosis or hospital discharge. Mortality and BSI recurrence were assessed at 30-days, with post-discharge follow-up done by reviewing medical records and national death registry [41].

Inclusion criteria for this study was adult individuals (≥ 18 years old) with BSI caused by *K. aerogenes*, *K. pneumoniae* or *E. cloacae*, each a different exposure group. Polymicrobial bacteraemia episodes were excluded. Primary outcome was a composite clinical outcome that includes all-cause mortality or recurrence within 30 days after BSI diagnosis. BSI recurrence was defined as bacteraemia with the same microorganism after negative blood cultures or clinical improvement and completion of active antimicrobial therapy [42]. Secondary outcomes were persistent fever, persistent BSI, and device infection secondary to BSI. Persistent fever was defined as fever ≥ 72 h in patients treated with an in vitro active antimicrobial drug [42]. Persistent BSI was defined as positive blood culture with the same microorganism after 72 h of active antibiotic therapy [42]. Secondary infection included endovascular or orthopaedic devices infection secondary to BSI [42].

Setting of BSI acquisition was considered nosocomial if BSI occurred more than 48 h after hospital admission. Episodes were considered HAI if any of the following was present: intravenous therapy, wound care or specialized nursing care at home, dialysis, radiotherapy or chemotherapy, two or more attendances to specialized outpatient clinic, residency in a nursing home or long term care facility in the 30 days before BSI; major surgery 30 days before BSI (90 days if implant); or hospitalization for more than 2 days in acute or chronic care hospital in the 90 days before BSI. Episodes that don’t fulfil these criteria were considered community acquired.

Date of blood sample collection was considered the start of follow-up. In individuals with a primary outcome event, end of follow-up was considered when one of the events from the composite outcome was reached (death or recurrence), whatever happens first. In individuals without primary outcome, the date of last assessment was considered as the end of follow-up. In case of missing data in this variable, the date of hospital discharge was considered. Follow-up was right censored at 30 days after blood culture collection, according to the original study protocol. Loss to follow-up was considered when there was neither the date of last assessment nor date of hospital discharge.

### Statistical analysis

Frequencies and proportions were used to describe the cohort characteristics and reported through tabulation of categorical variables by the exposure variable (*K. aerogenes, E. cloacae* and *K. pneumoniae*), including missing data. Median and interquartile range were calculated to describe non-normally distributed data and stratified by the microorganism group. All records in the final sample size for data analysis were included in the univariate and multivariate analysis, regardless of missing data in covariates.

To assess the impact of potential bias caused by excluding individuals with missing data on follow-up time, patients included in the final analysis were compared with the patients excluded. Differences in these groups were inspected and assessed using Pearson chi-squared test for categorical variables and Wilcoxon rank-sum test for continuous skewed variables (Additional file 1: Table S1).

Univariate analysis used two models: model 1 compares outcomes in *K. aerogenes and E. cloacae* groups with a baseline group of *K. pneumoniae*; model 2 compares outcomes in *K. aerogenes* and *K. pneumoniae* groups with a baseline group of *E. cloacae*. Rate of the outcome per 1000 persons-day and the 95% confidence interval for the exposure variable and other independent variables was calculated. Rate ratio and the 95% confidence interval of the association between the composite clinical outcome and the different microorganism’s groups and other independent variables was calculated using a multilevel mixed-effect Poisson regression model considering hospital as the first level, assuming clustering of observations within hospital. Wald test was used to test for association.

A direct acyclic diagram (DAG) was built to describe the conceptual framework between potential confounders and other independent variables in the association between exposure (*K. aerogenes*, *K. pneumoniae* or *E. cloacae* BSI) and outcome (death or recurrence), based on literature review (Additional file 1: Fig. S1). Possible confounders identified in the conceptual framework were BSI source, hospital ward, comorbidities, age, and antibiotic therapy. DAG was useful to exclude other independent variables from the multivariate model that can be in the causal pathway (severity) or ancestors of the exposure and/or outcome (BSI acquisition, antibiotic resistance, devices, and procedures). Disease severity is a mediator of the association between the exposure (microorganisms) and the outcome (death or recurrence), which may bias the results if included in the analysis. Ancestors are variables that are indirectly associated with the exposure or outcome through confounders; their inclusion in the model is redundant and can compromise model stability when missing data is an issue.

Multilevel mixed-effect Poisson regression model was used to calculate the crude and adjusted rate ratio for each possible confounder. Multicollinearity was checked by using variance inflation factor and variables with cut-off higher than 10 were excluded. The final multivariate model included all the variables without multicollinearity defined a priori as confounders (age and sex) and identified through the conceptual framework (BSI source, hospital ward, Charlson score and active antibiotic therapy).

## Results

### Descriptive analysis

From the 664 records in the PRO-BAC dataset with BSI caused by the microorganisms of interest, 132 were excluded due to exclusion criteria (131 polymicrobial BSI and 1 age < 18-year-old) and 89 were excluded due to missing data on follow-up time. The final sample size for analysis included 443 BSI episodes: 29 *K. aerogenes*, 77 *E. cloacae* and 337 *K. pneumoniae* (Additional file 1: Fig. S2).

The median follow-up time was 30 days (IQR 11, 71) and similar between exposure groups (*K. pneumoniae* 31 days vs *E. cloacae* 26 days vs. *K. aerogenes* 30 days) (Table 1).

**Table 1:** Sample characteristics: descriptive analysis according to microorganism group

Patient’s characteristics	*K. pneumoniae* (n = 337)	*E. cloacae* (n = 77)	*K. aerogenes* (n = 29)
Sex			
Female	134 (39.8%)	24 (31.2%)	8 (27.6%)
Missing	2 (0.6%)	0 (0.0%)	0 (0.0%)
Age*			
18–59	79 (23.4%)	23 (29.9%)	3 (10.3%)
60–69	93 (27.6%)	20 (26.0%)	9 (31.0%)
70–79	91 (27.0%)	21 (27.3%)	11 (37.9%)
≥ 80	74 (22.0%)	13 (16.9%)	6 (20.7%)
Charlson index*			
≥ 5	159 (47.2%)	34 (44.2%)	15 (51.7%)
Department responsible			
Medical	185 (54.9%)	38 (49.4%)	19 (65.5%)
Surgery	73 (21.7%)	22 (28.6%)	5 (17.2%)
ICU	42 (12.5%)	10 (13.0%)	3 (10.3%)
Other	29 (8.6%)	5 (6.5%)	2 (6.9%)
Missing	8 (2.4%)	2 (2.6%)	0 (0.0%)
Setting of BSI acquisition*			
Community	90 (26.7%)	8 (10.4%)	9 (31.0%)
Nosocomial	155 (46.0%)	55 (71.4%)	12 (41.4%)
HAI	92 (27.3%)	14 (18.2%)	8 (27.6%)
BSI source			
Abdominal	79 (23.4%)	21 (27.3%)	6 (20.7%)
Catheter	33 (9.8%)	14 (18.2%)	3 (10.3%)
Respiratory	21 (6.2%)	4 (5.2%)	3 (10.3%)
Urinary	141 (41.8%)	14 (18.2%)	11 (37.9%)
Other	8 (2.4%)	3 (3.9%)	0 (0.0%)
Unknown	54 (16.0%)	21 (27.3%)	6 (20.7%)
Missing	1 (0.3%)	0 (0.0%)	0 (0.0%)
Pitt score*			
≥ 4	35 (10.4%)	7 (9.1%)	2 (6.9%)
Severe sepsis*			
Yes	66 (19.6%)	11 (14.3%)	5 (17.2%)
Septic shock*			
Yes	51 (15.1%)	7 (9.1%)	5 (17.2%)
ESBL production			
Yes	79 (23.4%)	2 (2.6%)	0 (0.0%)
Missing	85 (25.2%)	23 (29.9%)	9 (31.0%)
Carbapenemase production			
Yes	33 (9.8%)	0 (0.0%)	1 (3.4%)
Missing	110 (32.6%)	27 (35.1%)	10 (34.5%)
Active empiric antibiotic			
Yes	250 (74.2%)	47 (61.0%)	19 (65.5%)
Missing	41 (12.2%)	14 (18.2%)	2 (6.9%)
Time to start ATB			
Before	59 (17.5%)	10 (13.0%)	3 (10.3%)
Same day	199 (59.1%)	47 (61.0%)	18 (62.1%)
≥ 1 days after	43 (12.8%)	9 (11.7%)	5 (17.2%)
Missing	36 (10.7%)	11 (14.3%)	3 (10.3%)
Composite outcome*	64 (19.0%)	16 (20.8%)	2 (6.9%)
Recurrence*	15 (4.5%)	8 (10.4%)	0 (0.0%)
All-cause mortality*	62 (18.4%)	15 (19.5%)	2 (6.9%)
Infection related mortality*	38 (11.3%)	7 (9.1%)	1 (3.4%)
Persistent BSI*	19 (5.6%)	5 (6.5%)	0 (0.0%)
Fever ≥ 72H*	49 (14.5%)	11 (14.3%)	5 (17.2%)
Device infection*	8 (2.4%)	1 (1.3%)	1 (3.4%)
Length of hospital stay	14 (7, 31) (n = 239)	21 (11, 35) (n = 54)	11 (4, 22) (n = 19)
Follow-up time, days	31 (11, 73) (n = 337)	26 (11, 52) (n = 77)	30 (11, 61) (n = 29)

Composite outcome: death or recurrence at 30-day. Severe sepsis and septic shock are mutually exclusive. Categorical variables reported as frequencies and proportions. Continuous variables reported as median, IQR and completed records

ATB: antibiotic; BSI: bloodstream infection; ESBL: extended-spectrum beta-lactamase; HAI: healthcare associated infection; ICU: intensive care unit

^*^No missing data

Comparing between groups, patients with *E. cloacae* BSI had more frequent nosocomial infections (*K. pneumoniae* 46.0%, n = 155 vs. *E. cloacae* 71.4%, n = 55 vs. *K. aerogenes* 41.4%, n = 12), less common BSI secondary to urinary source (*K. pneumoniae* 41.8%, n = 141 vs. *E. cloacae* 18.2%, n = 14 vs. *K. aerogenes* 37.9%, n = 11) and the length of hospital stay was longer (*K. pneumoniae* 14 days vs. *E. cloacae* 21 days vs. *K. aerogenes* 11 days); on the other hand, in *K. pneumoniae* group, antibiotic resistance was more common (ESBL: *K. pneumoniae* 23.4%, n = 79 vs. *E. cloacae* 2.6%, n = 2 vs. *K. aerogenes* 0%, n = 0; carbapenemase production: *K. pneumoniae* 8.8%, n = 33 vs. *E. cloacae* 0%, n = 0 vs. *K. aerogenes* 3.4%, n = 1) but active empiric antibiotic therapy more frequent (*K. pneumoniae* 74.2%, n = 250 vs. *E. cloacae* 61.0%, n = 47 vs. *K. aerogenes* 65.5%, n = 19) (Table 1).

The primary composite outcome was less frequent in *K. aerogenes* group (*K. pneumoniae* 19.0%, n = 64 vs. *E. cloacae* 20.8%, n = 16 vs. *K. aerogenes* 6.9%, n = 2) reflecting the findings of all-cause mortality (*K. pneumoniae* 18.4%, n = 62 vs. *E. cloacae* 19.5%, n = 15 vs. *K. aerogenes* 6.9%, n = 2) and BSI recurrence (*K. pneumoniae* 4.5%, n = 15 vs. *E. cloacae* 10.4%, n = 8 vs. *K. aerogenes* 0%, n = 0). Persistent BSI was also less frequent in *K. aerogenes* group, while other secondary outcomes were more common (Table 1).

Variables with more than 10% of missing data were ESBL production (26.4%, n = 117), carbapenemase production (33.2%, n = 147), active empiric antibiotic therapy (12.9%, n = 57) and time to start antibiotic (11.1%, n = 49) (Additional file 1: Table S1). Missing data were similar between groups, except in active empiric antibiotic therapy, in which the proportion of missing data was higher in the *E. cloacae* group (Table 1).

### Survival analysis

In total, 436 episodes were included in the survival analysis, corresponding to 75 events for a total time at risk of 9360 days (8 events per 1000 person-days). Seven BSI episodes (six *K. pneumoniae* and one *E. cloacae*) were excluded due to death at day 0.

**Fig. 1:**
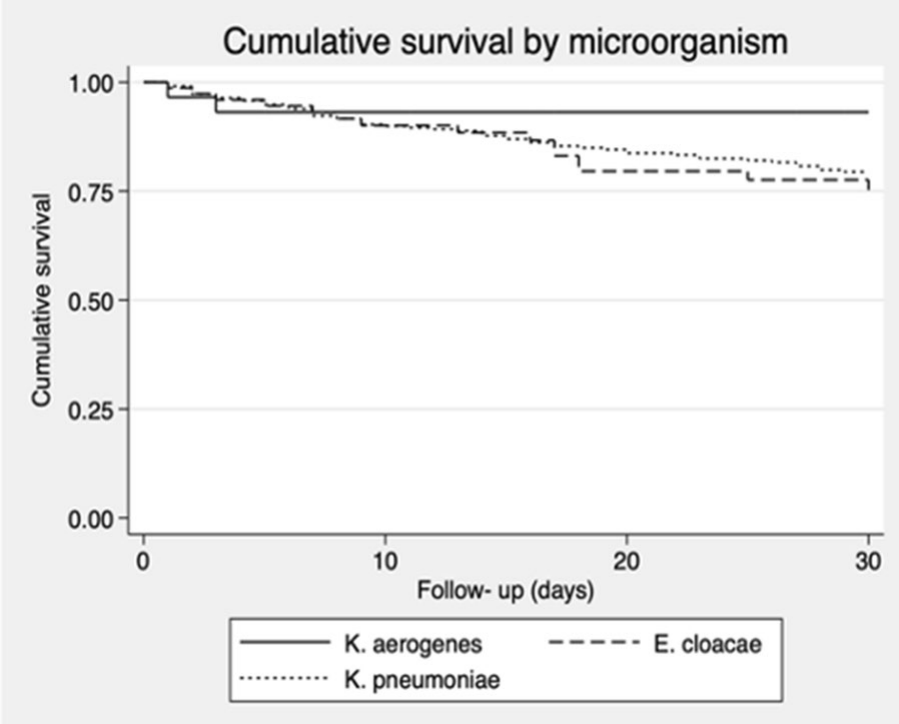
Kaplan–Meier curve: cumulative survival by microorganism

The Kaplan–Meier curve shows a similar survival in *E. cloacae* and *K. pneumoniae* groups until day 17 (around 87.5% survival). After day 17 the survival for *E. cloacae* group is slightly lower than in *K. pneumoniae* group, reaching around 75% in both groups at day 30. The *K. aerogenes* survival curve is difficult to interpret because only two events occurred in the early follow-up period (Fig. 1). There was no evidence of difference between groups in the log-rank test (p-value 0.337).

### Univariate analysis

Patients with *K. aerogenes* BSI were 58% and 65% less likely to die or have recurrence, when compared with *K. pneumoniae* (rate ratio (RR) 0.42; 95% CI 0.10 to 1.71) and *E. cloacae* (RR 0.35; 95% CI 0.08 to 1.55), respectively, without statistical evidence for an association between microorganisms and outcome (p-value 0.379) (Table 2). Individuals with *E. cloacae* BSI were 18% more likely of having the outcome of interest (RR 1.18; 95% CI 0.67 to 2.10), when compared to *K. pneumoniae* group, without statistical evidence of an association (p-value 0.379) (Table 2).

**Table 2:** Univariate analysis: association between the microorganism and other independent variables with composite outcome (death or recurrence)

Variables	Events	Persons-day	Rate per 1000 (95% CI)	Rate ratio (95% CI)*	p-value†
Microorganism					
* K. pneumoniae*	58	7196	8.06 (6.23–10.43)	Model 1‡: 1.00 Model 2§: 0.86 (0.48–1.50)	0.379
* E. cloacae*	15	1555	9.65 (5.82–16.00)	Model 1‡: 1.18 (0.67–2.10) Model 2§: 1.00	
* K. aerogenes*	2	609	3.28 (0.82–13.13)	Model 1‡: 0.42 (0.10–1.71) Model 2§: 0.35 (0.08–1.55)	
Sex					
Female	33	3285	10.05 (7.14–14.13)	1.00	0.110
Male	41	6028	6.80 (5.01–9.24)	0.69 (0.43–1.09)	
Age					
18–59	12	2425	4.95 (2.81–8.71)	1.00	0.039
60–69	16	2657	6.02 (3.69–9.83)	1.17 (0.55–2.49)	
70–79	29	2609	11.12 (7.72–16.00)	2.21 (1.12–4.36)	
≥ 80	18	1669	10.78 (6.79–17.12)	2.14 (1.02–4.46)	
Charlson index					
< 5	19	5149	3.69 (2.35–5.79)	1.00	0.001
≥ 5	56	4211	13.30 (10.23–17.28)	3.56 (2.11–6.01)	
Department responsible					
Medical	45	5132	8.77 (6.55–11.74)	1.00	0.198
Surgery	10	2145	4.66 (2.51–8.66)	0.53 (0.27–1.06)	
ICU	13	1168	11.13 (6.46–19.17)	1.22 (0.65–2.30)	
Other	4	717	5.58 (2.09–14.86)	0.66 (0.24–1.87)	
Setting of BSI acquisition					
Community	20	1994	10.03 (6.47–15.55)	1.00	0.258
Nosocomial	41	4947	8.29 (6.10–11.26)	0.79 (0.46–1.37)	
HAI	14	2419	5.79 (3.43–9.77)	0.56 (0.28–1.12)	
BSI source					
Abdominal	19	2245	8.46 (5.40–13.27)	1.00	0.598
Catheter	5	1156	4.33 (1.80–10.39)	0.51 (0.19–1.38)	
Respiratory	7	617	11.35 (5.41–23.80)	1.35 (0.56–3.24)	
Urinary	27	3401	7.94 (5.44–11.58)	0.93 (0.51–1.68)	
Other	3	226	13.27 (4.28–41.16)	1.60 (0.47–5.47)	
Unknown	13	1711	7.60 (4.41–13.09)	0.87 (0.43–1.77)	
Pitt score					
< 4	55	8647	6.36 (4.88–8.28)	1.00	0.001
≥ 4	20	713	28.06 (18.10–43.48)	4.47 (2.63–7.59)	
Severe sepsis					
No	51	7695	6.63 (5.04–8.72)	1.00	0.002
Yes	24	1665	14.41 (9.66–21.51)	2.16 (1.32–3.54)	
Septic shock					
No	53	8328	6.36 (4.86–8.33)	1.00	0.001
Yes	22	1032	21.32 (14.04–32.38)	3.45 (2.08–5.74)	
ESBL production					
No	37	5182	7.14 (5.17–9.85)	1.00	0.616
Yes	15	1737	8.64 (5.21–14.32)	1.17 (0.63–2.17)	
Carbapenemase production					
No	40	5501	7.27 (5.33–9.91)	1.00	0.894
Yes	5	704	7.10 (2.96–17.06)	1.07 (0.41–2.81)	
Active empiric antibiotic					
No	12	1523	7.88 (4.48–13.87)	1.00	0.863
Yes	50	6848	7.30 (5.53–9.63)	0.94 (0.49–1.81)	
Time to start ATB					
Before	16	1591	10.06 (6.16–16.42)	1.00	0.473
Same day	38	5747	6.61 (4.81–9.09)	0.69 (0.38–1.25)	
≥ 1 days after	9	1248	7.21 (3.75–13.86)	0.74 (0.32–1.70)	

ATB: antibiotic; BSI: bloodstream infection; ESBL: extended-spectrum beta-lactamase; HAI: healthcare associated infection; ICU: intensive care unit

^*^Multilevel mixed-effect Poisson regression estimate

^†^Wald test

^‡^Model 1 considering *K. pneumoniae* the baseline group

^§^Model 2 considering *E. cloacae* the baseline group

Men were 31% less likely to develop the composite outcome, when compared to women, without evidence of an association (RR 0.69; 95% CI 0.43 to 1.09; p-value 0.110) (Table 2). Compared to patients aged 18 to 59 years, individuals aged 70 to 79 years and over 80 years were 2.21 times (RR 2.21; 95% CI 1.12 to 4.36) and 2.14 times (RR 2.14; 95% CI 1.02 to 4.46) more likely to die or have recurrence, respectively, with evidence for this association (p-value 0.039) (Table 2).

There was evidence that participants with a Charlson comorbidity index ≥ 5 were 3.56 times more likely of dying or having recurrence, when compared to index < 5 (RR 3.56; 95% CI 2.11 to 6.01; p-value 0.001) (Table 2). Compared with individuals admitted to medical wards, patients in surgical departments were 47% less likely to die or recurrence (RR 0.53; 95% 0.27 to 1.06), without statistical evidence of an association (p-value 0.198) (Table 2). Individuals with catheter associated BSI were 49% less likely to have the composite outcome (RR 0.51; 95% CI 0.19 to 1.38), while respiratory tract were 35% more likely (RR 1.35; 95% CI 0.56 to 3.24), when compared to abdominal source, without statistical evidence of an association (p-value 0.598) (Table 2). There was no association between active empiric antibiotic and the outcome under study (RR 0.94; 95% CI 0.49 to 1.81, p-value 0.863) (Table 2).

### Multivariate analysis

In the adjusted analysis the association between microorganisms and clinical outcome was slightly decreased towards the null, when compared to the crude analysis. Individuals with *K. aerogenes* were 56% and 52% less likely to die or having recurrence, when compared to *K. pneumoniae* (RR 0.44; 95% CI 0.11 to 1.84; p-value 0.262) and *E. cloacae* (RR 0.48; 95% CI 0.10 to 2.30; p-value 0.361), respectively, without statistical evidence of an association (Table 3).

**Table 3:** Multivariate analysis adjusted for age, sex, Charlson score, department, BSI source, and active empiric antibiotic

Variables	Rate ratio (95% CI)*	p-value†
Microorganism		
* K. pneumoniae*	Model 1‡: 1.00 Model 2§: 1.10 (0.52–2.31)	Model 1‡: – Model 2§: 0.806
* E. cloacae*	Model 1‡: 0.91 (0.43–1.92) Model 2§: 1.00	Model 1‡: 0.806 Model 2§: –
* K. aerogenes*	Model 1‡: 0.44 (0.11–1.84) Model 2§: 0.48 (0.10–2.30)	Model 1‡: 0.262 Model 2§: 0.361
Age		
18–59	1.00	
60–69	0.73 (0.30–1.78)	0.483
70–79	1.32 (0.59–2.93)	0.499
≥ 80	0.98 (0.40–2.41)	0.959
Sex		
Female	1.00	
Male	0.76 (0.44–1.31)	0.330
Charlson index		
< 5	1.00	
≥ 5	3.87 (1.94–7.69)	< 0.001
Department responsible		
Medical	1.00	
Surgery	0.66 (0.29–1.52)	0.331
ICU	1.09 (0.52–2.31)	0.820
Other	0.86 (0.29–2.58)	0.794
BSI source		
Abdominal	1.00	
Catheter	0.91 (0.31–2.66)	0.863
Respiratory	1.39 (0.50–3.82)	0.527
Urinary	1.12 (0.56–2.25)	0.753
Other	1.94 (0.37–10.03)	0.431
Unknown	0.93 (0.40–2.18)	0.864
Active empiric antibiotic		
No	1.00	
Yes	0.84 (0.41–1.72)	0.634

Includes 375 observations, 25 hospitals

BSI: bloodstream infection; ICU: intensive care unit

^*^Multilevel mixed-effect Poisson regression estimate

^†^Chi-squared test

^‡^Model 1 considering *K. pneumoniae* the baseline group

^§^Model 2 considering *E. cloacae* the baseline group

Men were 24% less likely to develop the composite outcome, when compared to women (RR 0.76; 95% CI 0.44 to 1.31; p-value 0.330) (Table 3). Compared to the age group 18 to 59 years, individuals between 60 to 69 years were 27% less likely to die or have recurrence (RR 0.73; 95% CI 0.30 to 1.78; p-value 0.483), while individuals between 70 to 79 years old were 32% more likely (RR 1.32; 95% CI 0.59 to 2.93; p-value 0.499) (Table 3).

Charlson comorbidity index equal or above 5 was associated with a 3.87-fold increase in the risk of death or recurrence, when compared to patients with a lower index (RR 3.87; 95% CI 1.94 to 7.69; p < 0.001) (Table 3). Patients admitted to surgical had 34% less risk of worse outcome, when compared to individuals in the medical ward (RR 0.66; 95% CI 0.29 to 1.52; p-value 0.331) (Table 3). The risk of death or recurrence was 39% higher in BSI secondary to respiratory tract infection, when compared to abdominal source (RR 1.39; 95% CI 0.50 to 3.82; p-value 0.527) (Table 3). Patients treated with active empiric therapy seems to have a lower risk of death or recurrence, without statistical evidence of an association (RR 0.84; 95% CI 0.41 to 1.72; p-value 0.634) (Table 3).

## Discussion

In this cohort study, individuals with *K. aerogenes* BSI were 58% and 65% less likely to die or have recurrence, when compared to *K. pneumoniae* and *E. cloacae*, respectively, although there was no evidence for an association. After adjusting for possible confounders, the rate ratio decreased towards the null effect. Despite the structural and genetic similarities between *K. aerogenes* and *K. pneumoniae*, this study demonstrates that *K. aerogenes* infection might have better outcomes.

In the survival analysis *E. cloacae* and *K. pneumoniae* groups have similar survival proportion. In fact, in the univariate analysis, the difference between these pathogens was very small; patients with *E. cloacae* BSI were 18% more likely of dying or having recurrence, when compared to *K. pneumoniae* group, without statistical evidence of an association. In our study, patients with *E. cloacae* BSI had more frequent nosocomial infections and catheter-associated BSI, when compared to other groups, which might be indicators of worse prognosis in these patients.

Previous studies have found a trend in the opposite direction, with higher mortality in *K. aerogenes* when compared to *E. cloacae* BSI, without statistical evidence of difference between groups. [37, 40] A multicentre case–control study conducted in five Spanish hospitals have assessed all-cause mortality at 30-day follow-up in cases with *K. aerogenes* or *E. cloacae* BSI with a control group of individuals without BSI matched by age, sex, and hospital area. [37] The authors reported no difference in 30-day mortality between these two pathogens and *E. cloacae* and *K. aerogenes* groups were similar in terms of place of acquisition, BSI source, severity, and antibiotic resistance. [37] The higher mortality in this study can be due to higher proportion of nosocomial *K. aerogenes* infections. [37] A single centre cohort study in the USA compared in-hospital all-cause mortality in patients with *K. aerogenes* and *E. cloacae* BSI. [40] When assessing a composite outcome of death before discharge, recurrence and/or complications, *K. aerogenes* group had a worse clinical outcome, when compared to *E. cloacae* [40]. The worst clinical outcome in the *K. aerogenes* group can be due to higher proportion of acute kidney injury in these patients, an outcome that was not included in the PRO-BAC analysis [40]. Proportion of recurrence was very low in both groups, when compared to PRO-BAC results [40]. The single centre design and the long period of enrolment can justify this difference, for example through spread of more virulent clones in the USA centre, differences in healthcare services through time or higher severity of patients admitted to this institution. This study also has the limitations of not considering the differences in length of hospital stay and it is not clear if these events have occurred before or after 30-days follow-up. An older cohort study from 2010, compared mortality in individuals admitted to a single centre in Seoul with *K. aerogenes* and *E. cloacae* BSI [39]. In this Korean study, there was statistical evidence of a higher mortality in the *K. aerogenes* group at 7-, 14- and 21-days follow-up [39]. Even though the proportion of septic shock is similar in both studies, the differences in results can be due to a higher proportion of ESBL production and nosocomial infections in the *K. aerogenes* group in the Korean study, when compared to PRO-BAC dataset [39]. The single centre design is a limitation of this study that could have influenced the higher proportion of ESBL-producing bacteria and nosocomial infections, more common in centres with inefficient infection prevention and control measures [43, 44]. Additionally, the analysis was not adjusted for confounding and *K. aerogenes* group was more frequently treated with inappropriate therapy, which could have biased the results [39].

On the other hand, findings from a more recent Korean case–control study are similar to the PRO-BAC analysis; there was statistical evidence of a higher mortality in *E. cloacae* group, when compared to *K. aerogenes* in the propensity score matched analysis [38]. Compared to PRO-BAC cohort, patients in Korean study have more frequent healthcare-associated infections and resistance to 3rd generation cephalosporins, while a higher proportion was treated with appropriate empiric therapy [38]. Even though the single centre design could be a limitation to results generalizability, the propensity score matched analysis reduces bias due to confounding. We didn’t find any study comparing clinical outcomes in *K. aerogenes* with *K. pneumoniae* BSI.

Our study uses a composite outcome that includes mortality or BSI recurrence at 30-day follow-up which may be consider an advantage, allowing to reflect a wider spectrum of clinical outcomes and increase the statistical study power, by allowing a higher number of outcome events per exposure group [45, 46]. The use of conceptual framework to decide which potential confounders should be included in the adjusted analysis improved the reliability of results, by avoiding over adjusting, increases the transparency of the analysis and supports the interpretation of the study results [47, 48].

The main limitation of our study is the small sample size and the low number of events in the three pathogens groups (particularly in the *K. aerogenes* group). The outcome assessment was done by review of medical records and national death registry, so we don’t expect that misclassification of the outcome have been an important cause of bias. Additionally, seven observations were excluded from the analysis, due to death at day 0, which could represent severe ill patients infected by a more virulent pathogen. However, most of them were infected by *K. pneumoniae*, which might have biased the results towards the null. Another relevant weakness was the exclusion of a high number of BSI episodes that didn’t have the date of entry or exit in the study. When comparing patients included and excluded from the analysis, the groups are very similar, only different in terms of antibiotic resistance and comorbidities (Additional file 1: Table S1). The impact of these differences is difficult to predict; patients excluded could be less severe due to lower proportion of comorbidities or more severe due to higher presence of carbapenemase.

Reporting the results using rate per 1000 person-days provides an adjusted analysis for the time at risk. The fact that the analysis didn’t take into consideration the possibility of competing events probably hasn’t affected the results because the overall mortality in PRO-BAC dataset is lower than in previous studies.

Due to small sample size this study results might not be generalizable. Nevertheless, the high proportion of death and recurrence in general in *K. aerogenes*, *E. cloacae* and *K. pneumoniae* BSI, should raising awareness about the burden of these pathogens and the importance of appropriate therapy. The study findings stress the need to develop and implement rapid diagnostic tools in BSI, which could help in early prescription of appropriate target therapy. Other strategies, for example, antimicrobial stewardship programs, can also be important to support clinicians when deciding empirical therapy, taking into consideration local epidemiological data.

## Conclusions

This study suggests that BSI caused by *K. aerogenes* may have a better prognosis than *E. cloacae* or *K. pneumonia*; and that *E. cloacae* BSI may have a similar prognosis as *K. pneumoniae*.

New studies with bigger sample size and focused on other populations (e.g., paediatrics) will be useful to clarify the contradictory findings described in literature. It would be interesting to have more studies using composite outcomes, since they represent a wider variety of possible outcomes, reflecting the natural history of this disease.

### Supplementary Information

**Additional file 1: Figure S1. **Directed acyclic diagram of potential confounders, effect modifiers and other independent variables in the association between *K. aerogenes*, *K. pneumoniae* or *E. cloacae *BSI and clinical outcome (death or recurrence). **Figure S2.** Inclusion flow diagram for data analysis. **Table S1.** Characteristics of total sample, episodes included and excluded in analysis.

## Data Availability

The datasets used and/or analysed during the current study are available from the corresponding author on reasonable request.

## Abbreviations

AMS/IPC: Antimicrobial stewardship and infection prevention and control
ATB: Antibiotic
BSI: Bloodstream infection
CI: Confidence interval
DAG: Directed acyclic diagram
ESBL: Extended spectrum beta-lactamase
HAI: Healthcare-associated infection
ICU: Intensive care unit
KPC: *Klebsiella pneumoniae* carbapenemase
MDR: Multidrug resistance
