# Supplementary material for: Differences in clinical outcomes of bloodstream infections caused by Klebsiella aerogenes, Klebsiella pneumoniae and Enterobacter cloacae: a multicentre cohort study
Source: Ann Clin Microbiol Antimicrob. 2024 May 6;23:42. doi: 10.1186/s12941-024-00700-8 (PMC11071190; doi:10.1186/s12941-024-00700-8)
Supplement: Supplementary file 1 — Additional file 1: Figure S1. Directed acyclic diagram of potential confounders, effect modifiers and other independent variables in the association between K. aerogenes, K. pneumoniae or E. cloacae BSI and clinical outcome (death or recurrence). Figure S2. Inclusion flow diagram for data analysis. Table S1. Characteristics of total sample, episodes included and excluded in analysis. [file 12941_2024_700_MOESM1_ESM.docx]

**Supplementary material “Differences in clinical outcomes of bloodstream infections caused by *Klebsiella aerogenes*, *Klebsiella pneumoniae* and *Enterobacter cloacae*: a multicenter cohort study”**

**Figure S1 – Directed acyclic diagram of potential confounders, effect modifiers and other independent variables in the association between *K. aerogenes*, *K. pneumoniae* or *E. cloacae* BSI and clinical outcome (death or recurrence).**


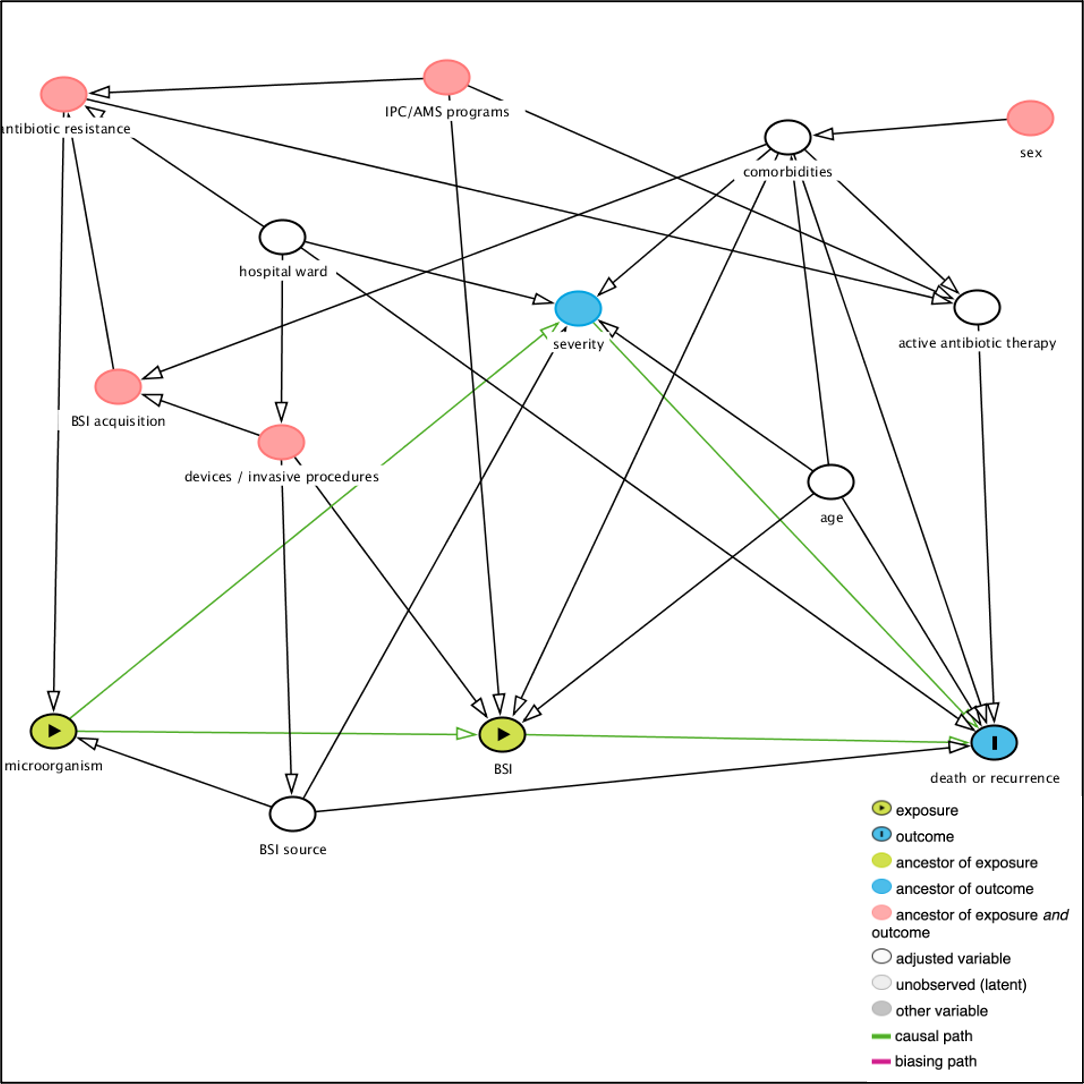


Figure description: The diagram is a graphic representation of potential confounders and effect modifiers in the casual pathway between the exposure “BSI caused by *K. aerogenes*, *K. pneumoniae* and *E. cloacae*” (in green) and the composite outcome “death or recurrence” (in blue). Variables in white are considered possible confounders (included in the adjusted analysis), while variables in pink are considered ancestor of exposure and/or outcome (not included in the analysis). Causal pathway is represented with green connections. Severity is a mediator in the causal pathway between the exposure and the outcome.

Acronyms: AMS – antimicrobial stewardship; BSI – bloodstream infection; IPC – infection prevention and control.

Notes: Directed acyclic diagram created in DAGitty online. ^1^

**Figure S2 – Inclusion flow diagram for data analysis**


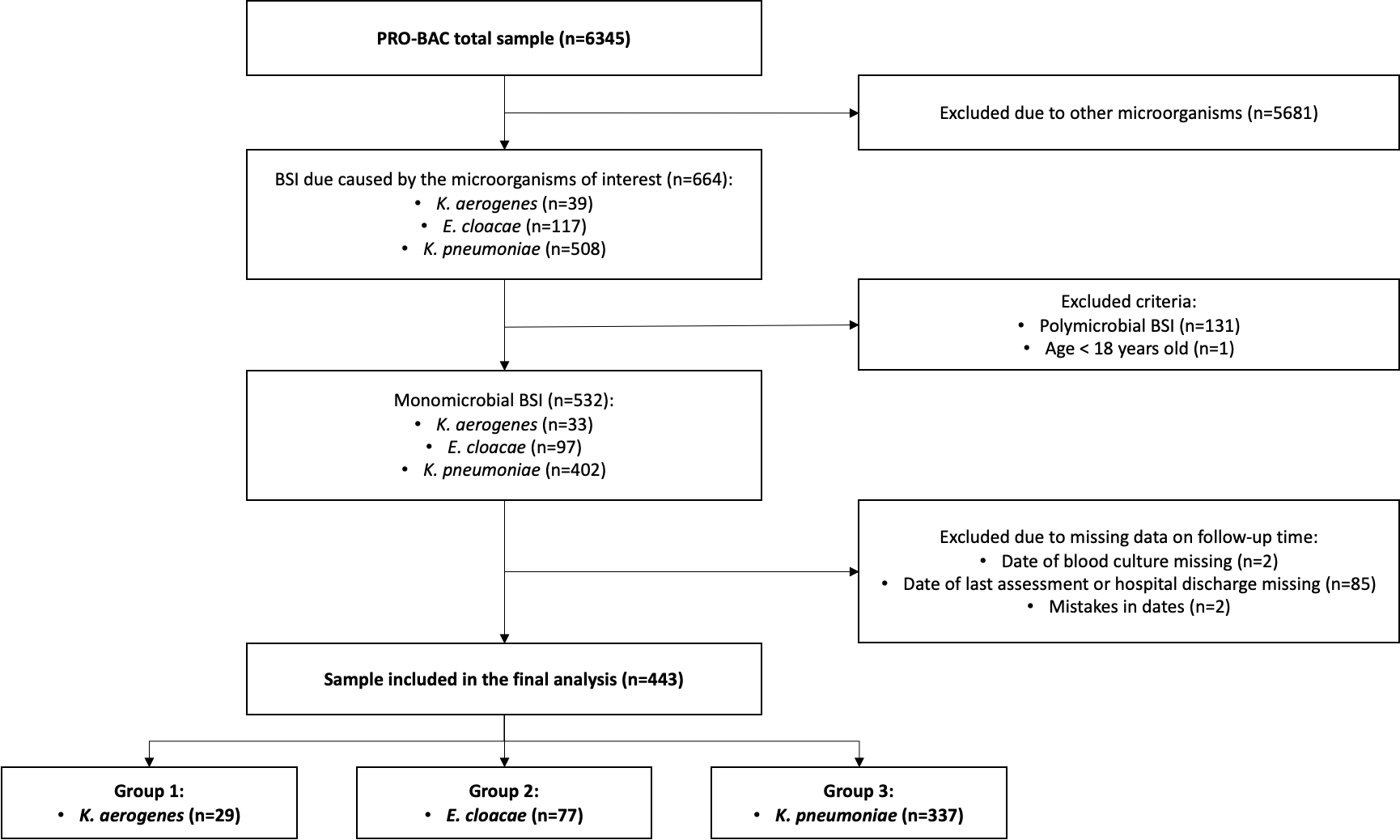


## **Table S1 – Characteristics of total sample, episodes included and excluded in analysis.**

| **Variables** | | **Total sample (532)** | **Included (n=443)** | **Excluded (n=89)** | **p-value** |
| --- | --- | --- | --- | --- | --- |
| **Microorganism** | *K. pneumoniae* | 402 (75.6%) | 337 (76.1%) | 65 (73.0%) | 0.440* |
|  | *E. cloacae* | 97 (18.2%) | 77 (17.4%) | 20 (22.5%) |  |
|  | *K. aerogenes* | 33 (6.2%) | 29 (6.5%) | 4 (4.5%) |  |
| **Sex** | Female | 197 (37.0%) | 166 (37.5%) | 31 (34.8%) | 0.720* |
|  | Missing | 4 (0.8%) | 2 (0.5%) | 2 (2.2%) |  |
| **Age** | 18 - 59 | 123 (23.1%) | 105 (23.7%) | 18 (20.2%) | 0.820* |
|  | 60 - 69 | 145 (27.3%) | 122 (27.5%) | 23 (25.8%) |  |
|  | 70 - 79 | 147 (27.6%) | 123 (27.8%) | 24 (27.0%) |  |
|  | ≥ 80 | 115 (21.6%) | 93 (21.0%) | 22 (24.7%) |  |
|  | Missing | 2 (0.4%) | 0 (0.0%) | 2 (2.2%) |  |
| **Charlson index** | ≥ 5 | 236 (44.4%) | 208 (47.0%) | 28 (31.5%) | 0.011* |
|  | Missing | 2 (0.4%) | 0 (0.0%) | 2 (2.2%) |  |
| **Department responsible** | Medical | 281 (52.8%) | 242 (54.6%) | 39 (43.8%) | 0.430* |
|  | Surgery | 116 (21.8%) | 100 (22.6%) | 16 (18.0%) |  |
|  | ICU | 69 (13.0%) | 55 (12.4%) | 14 (15.7%) |  |
|  | Other | 45 (8.5%) | 36 (8.1%) | 9 (10.1%) |  |
|  | Missing | 21 (3.9%) | 10 (2.3%) | 11 (12.4%) |  |
| **Setting of BSI acquisition** | Community | 136 (25.6%) | 107 (24.2%) | 29 (32.6%) | 0.120* |
|  | Nosocomial | 256 (48.1%) | 222 (50.1%) | 34 (38.2%) |  |
|  | HAI | 135 (25.4%) | 114 (25.7%) | 21 (23.6%) |  |
|  | Missing | 5 (0.9%) | 0 (0.0%) | 5 (5.6%) |  |
| **BSI source** | Abdominal | 131 (24.6%) | 106 (23.9%) | 25 (28.1%) | 0.540* |
|  | Catheter | 55 (10.3%) | 50 (11.3%) | 5 (5.6%) |  |
|  | Respiratory | 34 (6.4%) | 28 (6.3%) | 6 (6.7%) |  |
|  | Urinary | 195 (36.7%) | 166 (37.5%) | 29 (32.6%) |  |
|  | Other | 14 (2.6%) | 11 (2.5%) | 3 (3.4%) |  |
|  | Unknown | 93 (17.5%) | 81 (18.3%) | 12 (13.5%) |  |
|  | Missing | 10 (1.9%) | 1 (0.2%) | 9 (10.1%) |  |
| **Pitt score**‡ | ≥ 4 | 48 (9.0%) | 44 (9.9%) | 4 (4.5%) | 0.100* |
| **Severe sepsis**‡ | Yes | 96 (18.0%) | 82 (18.5%) | 14 (15.7%) | 0.530* |
| **Septic shock**‡ | Yes | 74 (13.9%) | 63 (14.2%) | 11 (12.4%) | 0.640* |
| **ESBL production** | Yes | 95 (17.9%) | 81 (18.3%) | 14 (15.7%) | 0.170* |
|  | Missing | 166 (31.2%) | 117 (26.4%) | 49 (55.1%) |  |
| **Carbapenemase production** | Yes | 43 (8.1%) | 34 (7.7%) | 9 (10.1%) | 0.011* |
|  | Missing | 203 (38.2%) | 147 (33.2%) | 56 (62.9%) |  |
| **Active empiric antibiotic** | Yes | 351 (66.0%) | 316 (71.3%) | 35 (39.3%) | 0.220* |
|  | Missing | 99 (18.6%) | 57 (12.9%) | 42 (47.2%) |  |
| **Time to start ATB** | Before | 83 (15.6%) | 72 (16.3%) | 11 (12.4%) | 0.810* |
|  | Same day | 296 (55.6%) | 264 (59.6%) | 32 (36.0%) |  |
|  | ≥ 1 days after | 66 (12.4%) | 58 (13.1%) | 8 (9.0%) |  |
|  | Missing | 87 (16.4%) | 49 (11.1%) | 38 (42.7%) |  |
| **Composite outcome**‡ | | 105 (19.7%) | 101 (22.8%) | 4 (4.5%) | <0.001* |
| **Recurrence**‡ | | 23 (4.3%) | 23 (5.2%) | 0 (0.0%) | 0.028* |
| **All-cause mortality**‡ | | 83 (15.6%) | 79 (17.8%) | 4 (4.5%) | 0.002* |
| **Infection related mortality**‡ | | 49 (9.2%) | 46 (10.4%) | 3 (3.4%) | 0.037* |
| **Persistent BSI**‡ | | 26 (4.9%) | 24 (5.4%) | 2 (2.2%) | 0.210* |
| **Fever ≥ 72H**‡ | | 73 (13.7%) | 65 (14.7%) | 8 (9.0%) | 0.150* |
| **Device infection**‡ | | 12 (2.3%) | 10 (2.3%) | 2 (2.2%) | 1.000* |
| **Length of hospital stay** | | 14 (6, 29.5) (n=328) | 15 (7, 30) (n=317) | 0 (0, 0) (n=11) | <0.001† |
| **Follow-up time, days** | | 30 (11, 68) (n=445) | 30 (11, 71) (n=443) | -5.5 (-8, -3) (n=2) | 0.015† |

Acronyms: ATB – antibiotic; BSI – bloodstream infection; ESBL – extended-spectrum beta-lactamase; HAI – healthcare associated infection; ICU – intensive care unit.

Legend: * Pearson's chi-squared; † Wilcoxon rank-sum; ‡ No missing data.

Notes: Composite outcome: death or recurrence at 30-day. Categorical variables reported as frequencies and proportions. Continuous variables reported as median and IQR.

**References**

1. Textor J, van der Zander B, Gilthorpe MS, Liśkiewicz M, Ellison GT. Robust causal inference using directed acyclic graphs: the R package ‘dagitty’. *International Journal of Epidemiology* 2016; **45**: 1887–94.
